# An Explainable AI Framework for Corneal Imaging Interpretation and Refractive Surgery Decision Support

**DOI:** 10.3390/bioengineering12111174

**Published:** 2025-10-28

**Authors:** Mini Han Wang

**Affiliations:** 1 Zhuhai People’s Hospital (The Affiliated Hospital of Beijing Institute of Technology, Zhuhai Clinical Medical College of Jinan University), Zhuhai 519000, China; 1155187855@link.cuhk.edu.hk; 2 The Department of Ophthalmology and Visual Sciences, The Chinese University of Hong Kong, Hong Kong 999077, China; 3 Zhuhai Institute of Advanced, Technology Chinese Academy of Sciences, Zhuhai 519000, China

**Keywords:** explainable artificial intelligence (XAI), corneal bioengineering, neuro-symbolic techniques, large language model (LLM), corneal topography, surgical decision support

## Abstract

This study introduces an explainable neuro-symbolic and large language model (LLM)-driven framework for intelligent interpretation of corneal topography and precision surgical decision support. In a prospective cohort of 20 eyes, comprehensive IOLMaster 700 reports were analyzed through a four-stage pipeline: (1) automated extraction of key parameters—including corneal curvature, pachymetry, and axial biometry; (2) mapping of these quantitative features onto a curated corneal disease and refractive-surgery knowledge graph; (3) Bayesian probabilistic inference to evaluate early keratoconus and surgical eligibility; and (4) explainable multi-model LLM reporting, employing DeepSeek and GPT-4.0, to generate bilingual physician- and patient-facing narratives. By transforming complex imaging data into transparent reasoning chains, the pipeline delivered case-level outputs within ~95 ± 12 s. When benchmarked against independent evaluations by two senior corneal specialists, the framework achieved 92 ± 4% sensitivity, 94 ± 5% specificity, 93 ± 4% accuracy, and an AUC of 0.95 ± 0.03 for early keratoconus detection, alongside an F1 score of 0.90 ± 0.04 for refractive surgery eligibility. The generated bilingual reports were rated ≥4.8/5 for logical clarity, clinical usefulness, and comprehensibility, with representative cases fully concordant with expert judgment. Comparative benchmarking against baseline CNN and ViT models demonstrated superior diagnostic accuracy (AUC = 0.95 ± 0.03 vs. 0.88 and 0.90, *p* < 0.05), confirming the added value of the neuro-symbolic reasoning layer. All analyses were executed on a workstation equipped with an NVIDIA RTX 4090 GPU and implemented in Python 3.10/PyTorch 2.2.1 for full reproducibility. By explicitly coupling symbolic medical knowledge with advanced language models and embedding explainable artificial intelligence (XAI) principles throughout data processing, reasoning, and reporting, this framework provides a transparent, rapid, and clinically actionable AI solution. The approach holds significant promise for improving early ectatic disease detection and supporting individualized refractive surgery planning in routine ophthalmic practice.

## 1. Introduction

Corneal topography [1] is an essential imaging modality for evaluating the morphology and optical characteristics of the cornea. It provides high-resolution curvature, elevation, and thickness maps [2] that are indispensable for screening and diagnosing conditions [3] such as keratoconus [4], assessing suitability for refractive surgery, monitoring post-operative ectasia, and guiding the fitting of rigid gas-permeable or orthokeratology contact lenses [5]. However, the clinical interpretation of corneal topography remains largely dependent on human expertise. Manual analysis of its complex color-coded maps and derived indices can be time-consuming and prone to inter-observer variability, particularly when subtle early-stage abnormalities or multifactorial surgical risks are involved.

With the rapid development of artificial intelligence (AI) [6], deep learning models [7] have demonstrated remarkable performance in detecting keratoconus [8], predicting ectasia risk, and classifying corneal diseases. Yet, most current AI approaches function as “black boxes” [9], offering limited transparency regarding their reasoning process. This lack of interpretability hinders clinical trust and regulatory approval. At the same time, large language models (LLMs) [10], such as GPT-based systems [11], excel in natural language understanding and interactive report generation [12], but are not inherently designed to process high-dimensional corneal topography images or perform structured medical reasoning.

Neuro-symbolic techniques [13] provide a promising solution by bridging sub-symbolic neural perception [14] and symbolic logical reasoning [15]. They enable the transformation of continuous image features into discrete, clinically meaningful symbols [16] and support rule-based inference consistent [17] with ophthalmic knowledge. Integrating this paradigm with domain-specialized LLMs [18] offers a powerful way to achieve both precision and explainability in corneal topography analysis [19]. By coupling image-derived symbolic representations with knowledge graphs [20] and chain-of-thought prompts [21], the combined system can produce transparent, patient-tailored decision support and generate comprehensible reports for both clinicians and patients [22].

In this study, we propose a hybrid framework that integrates Neuro-Symbolic techniques with ophthalmology-focused LLMs to enable intelligent interpretation of corneal topography reports. Our method consists of four key modules: (1) construction of a corneal disease and surgery knowledge graph with standardized morphological symbols, (2) symbolic neural reasoning that converts multimodal corneal images into logical patterns, (3) medical LLM-based natural language interpretation using models [6] of DeepSeek [23] and GPT-4.0 [24,25], and (4) rigorous system validation on longitudinal clinical data. By combining explainable feature extraction, symbolic reasoning, and interactive language generation, this research aims to enhance early keratoconus detection, optimize surgical planning, and deliver understandable, personalized information to both doctors and patients. The proposed framework not only addresses the limitations of current black-box AI solutions but also contributes to the broader goal of safe and trustworthy AI in ophthalmology. Furthermore, the proposed Neuro-Symbolic + LLM framework uniquely integrates deep visual encoders with symbolic knowledge and Bayesian reasoning, enabling causal traceability and dual-audience (physician/patient) explainability—features absent in current keratoconus and refractive-surgery AI systems.

## 2. Materials and Methods

### 2.1. Study Design and Ethical Approval

This study was designed as a prospective observational investigation aimed at developing and validating an intelligent framework for the interpretation of corneal topography reports. All study procedures strictly adhered to the principles of the Declaration of Helsinki (2013 revision) and to Chinese national regulations on biomedical research involving human participants.

The complete research protocol, including participant recruitment, imaging procedures, data management, and AI algorithm development, underwent independent ethical review and approval by the Ethics Committee of Zhuhai People’s Hospital (approval number: [2024]-KT-67). Before any data collection, written informed consent was obtained from every participant after full explanation of the study objectives, procedures, potential risks, and data privacy protections.

**Figure 1:**
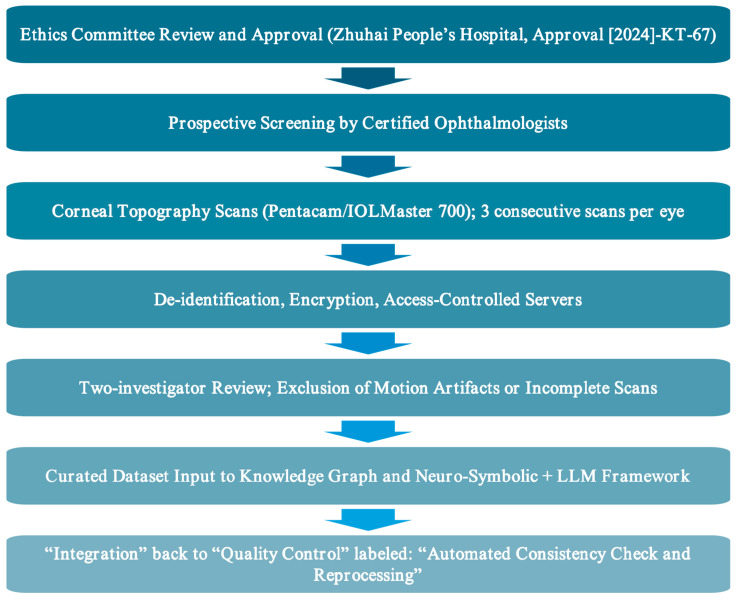
Ethical and Data-Handling Workflow for Corneal Topography AI Study.

As Figure 1 shows, flow diagram depicting the ethical approval, participant recruitment, data acquisition, anonymization, storage, quality control, and integration steps of the proposed Neuro-Symbolic + LLM corneal topography framework. All procedures adhered to institutional guidelines and the Declaration of Helsinki (2013 revision). The diagram highlights secure and standardized handling of patient data, ensuring ethical compliance and reproducibility.

All AI analyses were performed on a high-performance workstation equipped with an NVIDIA RTX 4090 GPU, 128 GB RAM, and an Intel i9 CPU, running Ubuntu 22.04. The computational pipeline was implemented in Python 3.10, using PyTorch 2.2.1 for deep learning model development, Pyro 1.8 for Bayesian probabilistic reasoning, and HuggingFace Transformers 4.42 for large language model integration. This environment ensured efficient handling of high-resolution corneal topography maps, symbolic reasoning computations, and natural-language report generation.

The CNN backbone for local feature extraction consisted of three convolutional blocks, each comprising a convolution layer followed by batch normalization and ReLU activation, with 3 × 3 kernels and max pooling after each block. The network was trained using the Adam optimizer with a learning rate of 1 × 10^−3^, a batch size of 16, and 120 epochs, incorporating a weight decay of 1 × 10^−5^ to reduce overfitting.

The Vision Transformer backbone for global contextual modeling comprised eight transformer layers with a hidden dimension of 512, a patch size of 16 × 16, and eight attention heads. Training employed the AdamW optimizer with a learning rate of 5 × 10^−4^, a batch size of 16, 120 epochs, and weight decay of 0.01.

For the Bayesian symbolic reasoning layer, Gaussian priors were applied over the distributions of symbolic variables, with a confidence threshold of 0.75 for disease inference. This configuration enabled rigorous probabilistic reasoning on top of neural feature representations, ensuring both robust diagnostic predictions and traceable decision pathways. Collectively, these specifications provide full reproducibility of the proposed Neuro-Symbolic + LLM framework, encompassing neural perception, probabilistic reasoning, and downstream natural-language interpretation.

Special attention was given to data confidentiality and cybersecurity: all identifiable patient information was removed or anonymized before algorithm training and analysis; electronic files were stored on encrypted, access-controlled servers in accordance with the hospital’s information security standards. The ethical approval covered all aspects of image acquisition, follow-up visits, multi-modal data integration, and subsequent symbolic-neural and large language model (LLM) analyses.

This comprehensive ethical framework ensures that the study meets international clinical research standards, safeguards participant rights and privacy, and provides a robust regulatory basis for the clinical application and potential commercialization of the proposed AI-assisted corneal topography decision-support system.

### 2.2. Participants and Data Acquisition

Participants were prospectively recruited from the Department of Ophthalmology at Zhuhai People’s Hospital and Zhuhai Aier Eye Hospital between January 2024 and December 2025. All volunteers were consecutively screened by certified ophthalmologists. Both eyes of each participant were examined; however, to avoid inter-eye correlation only one eye per participant was randomly selected for final analysis. The study aimed to include a diverse range of corneal conditions, encompassing healthy eyes, eyes with suspected or confirmed keratoconus, and eyes that had undergone previous refractive surgery.

Inclusion criteria required participants to be 18 to 65 years of age, capable of understanding the study objectives, and willing to provide written informed consent and attend at least one follow-up examination. Eligible subjects had to present either (1) a normal cornea without ocular disease or surgical history, (2) suspected or clinically diagnosed keratoconus at any stage, or (3) stable post-refractive-surgery status (LASIK, PRK, or SMILE) for at least six months. These criteria ensured that the dataset represented a broad spectrum of normal and pathological corneal morphologies.

Exclusion criteria were strictly applied to maintain image quality and patient safety. Individuals were excluded if they had a history of ocular trauma, infectious keratitis, active ocular inflammation, corneal scarring, opacity, or degeneration that could obscure imaging. Additional exclusions included severe dry eye disease or other ocular surface abnormalities that interfere with corneal shape measurement, systemic conditions known to affect corneal biomechanics such as connective tissue disorders or poorly controlled diabetes, and current or recent (within two weeks) contact lens wear. Participants unable to maintain stable fixation during imaging or to complete the scheduled follow-up were also excluded.

All enrolled subjects underwent comprehensive ophthalmic assessment, including best-corrected visual acuity, slit-lamp biomicroscopy, and intraocular pressure measurement, followed by corneal topography with a Pentacam HR (Oculus Optikgeräte GmbH, Wetzlar, Germany). Each eye was scanned three consecutive times by experienced technicians under mesopic conditions, and only datasets meeting the built-in Pentacam “OK” quality specification were retained. From each high-quality scan, curvature maps (anterior and posterior), elevation maps (anterior and posterior), pachymetry maps, and key diagnostic indices such as Kmax, minimum pachymetry, and Belin/Ambrosio Enhanced Ectasia Display (BAD-D) values were exported in raw, high-resolution formats.

All collected data were de-identified immediately after acquisition and stored on encrypted, password-protected hospital servers. A two-investigator quality-control process was applied to eliminate files with motion artifacts, incomplete coverage, or other technical defects. The curated and anonymized dataset was subsequently used to construct the corneal disease and surgery knowledge graph and to train and evaluate the Neuro-Symbolic techniques and large language model components of the proposed intelligent interpretation system.

### 2.3. Construction of Corneal Symbolic Knowledge Graph

All quantitative biometric parameters extracted from the IOLMaster 700 reports—including axial length (AL), anterior chamber depth (ACD), lens thickness (LT), anterior corneal curvature (K1/K2), total keratometry (TK1/TK2), and central corneal thickness (CCT)—were seamlessly integrated into the corneal disease and surgery knowledge graph. This graph encodes hierarchical relationships among anatomical structures, physiological norms, and pathological changes, allowing the continuous measurements to be translated into structured medical knowledge.

Within this framework, the raw numeric outputs were automatically transformed into clinically meaningful symbolic descriptors. Central steepening (defined by focal curvature elevation above 47 diopters), posterior elevation spike (exceeding established Belin/Ambrosio thresholds), normal central thickness (CCT between 500–560 µm), and age-related crystalline lens (derived from AL–ACD–LT relationships). Each symbol was linked to explicit decision rules in the knowledge graph, ensuring that subsequent diagnostic reasoning could be traced back to objective, well-defined criteria.

The symbolic reasoning engine then traversed the graph to generate transparent inference chains. For instance, a combination of “inferior–superior asymmetry” and “posterior elevation spike” activated a rule set pointing toward early keratoconus, while “normal central thickness” and “regular anterior curvature” supported a diagnosis of physiologic cornea. Such stepwise, machine-readable reasoning provides clinicians with an audit trail from raw data to diagnostic conclusion, overcoming the “black box” limitation of conventional deep learning.

**Figure 2:**
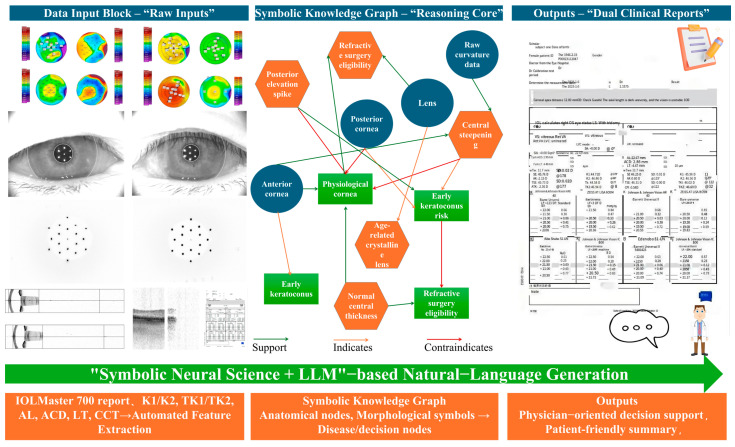
End-to-End Pipeline of the Neuro-Symbolic techniques + Large Language Model Framework for Intelligent Corneal Topography Interpretation.

An illustrative workflow is provided in Figure 2, where a representative subject’s measurements are depicted as graph nodes and logical edges. This visualization highlights how the system links multiple biometric features to higher-order disease concepts and ultimately to surgical decision-making. Through this integration of precise measurements and formal medical knowledge, the symbolic reasoning module ensures that every diagnostic output is both quantitatively grounded and clinically interpretable.

The symbolic thresholds used for classifying corneal curvature, pachymetry, and posterior elevation were established through a hybrid expert–data-driven process. Initial threshold ranges (e.g., Kmax > 47.2 D, thinnest pachymetry < 490 µm, posterior elevation > +15 µm) were derived from the Global Consensus on Keratoconus and Ectatic Diseases and refined in consultation with two senior corneal specialists. These expert-defined rules were subsequently validated on the training subset of the dataset, where iterative statistical calibration was applied to optimize agreement with ground-truth clinical labels while maintaining interpretability. This process ensured that all symbolic boundaries were both clinically meaningful and empirically justified.

### 2.4. Neuro-Symbolic Technique-Based Image Reasoning

To transform raw corneal topography data into interpretable diagnostic evidence, we developed a Neuro-Symbolic technique-based reasoning framework that tightly couples deep neural perception with symbolic logic. The workflow comprised three main components: multimodal feature extraction, symbolic projection, and probabilistic reasoning.

High-resolution corneal topography datasets—including curvature maps, anterior and posterior elevation maps, and pachymetry (thickness) distributions—were first standardized to a common polar coordinate system centered on the corneal apex. A deep neural architecture combining a convolutional neural network (CNN) backbone for local texture capture and a Vision Transformer (ViT) module for global context modeling was trained to learn multiscale spatial representations. The network output a latent feature vector encoding shape irregularity, thickness gradients, and biomechanical cues such as asymmetric steepening or focal thinning. Data augmentation (rotations, flips, and illumination normalization) and five-fold cross-validation were employed to enhance robustness despite the relatively small sample size.

The neural feature extraction backbone of the proposed framework integrates a convolutional neural network (CNN) and a Vision Transformer (ViT) to jointly capture local and global spatial features from corneal topography maps. The CNN encoder consists of three sequential Conv–BN–ReLU blocks with 3 × 3 kernels and max-pooling operations, optimized to extract fine-grained curvature and pachymetry patterns. The ViT module comprises eight transformer layers with a hidden dimension of 512, a patch size of 16 × 16, and eight attention heads to model long-range contextual dependencies across the corneal surface. Both networks were trained for 120 epochs with a batch size of 16, using the Adam optimizer (learning rate = 1 × 10^−3^, weight decay = 1 × 10^−5^) for the CNN and the AdamW optimizer (learning rate = 5 × 10^−4^, weight decay = 0.01) for the ViT. Gaussian priors were applied within the Bayesian reasoning layer, with a confidence threshold of 0.75 to determine disease inference. This configuration balances convergence stability, computational efficiency, and generalization performance, ensuring reproducibility across different experimental setups.

The continuous feature vectors were then mapped to the symbolic corneal knowledge graph constructed in Section 2.3. A learned embedding alignment module performed soft matching between latent neural features and predefined medical symbols (e.g., “central steepening,” “posterior elevation spike,” “inferior–superior asymmetry”). This mapping converted quantitative outputs into discrete, semantically meaningful predicates, effectively creating a symbolic representation of each patient’s corneal state.

On top of the symbolic representation, a probabilistic logic layer executed reasoning tasks that mimic expert clinical decision-making. The layer integrated symbolic reasoning (first-order logic rules)—such as “if central steepening and posterior elevation spike, then suspect early keratoconus”—with Bayesian inference to handle uncertainty and inter-feature variability. The combined engine generated transparent diagnostic hypotheses, graded keratoconus stages, and quantified risks of post-refractive-surgery ectasia.

The final symbolic reasoning outputs, including diagnostic confidence scores and recommended risk categories, were structured in a standardized JSON schema and fed directly to the LLM interpretation pipeline (Section 2.5). This ensured seamless interaction between image-derived symbols and the natural-language decision-support reports presented to clinicians and patients.

By uniting neural perception with formal symbolic logic, the proposed method preserves the high sensitivity of deep learning while providing clinically traceable reasoning chains, thereby enhancing trust and facilitating clinical adoption of AI-assisted corneal topography interpretation.

Figure 2 provides a comprehensive visual overview of the proposed AI workflow, which proceeds from raw clinical data to fully interpreted, patient- and physician-oriented reports. The figure is arranged from left to right to emphasize the sequential transformation of data into medical reasoning and decision support.

The left panel represents the data input block. Multi-page PDF reports generated by the IOLMaster 700 corneal topographer supply high-resolution curvature, elevation, and pachymetry maps together with key biometric measurements such as anterior corneal curvature (K1/K2), total keratometry (TK1/TK2), axial length (AL), anterior chamber depth (ACD), lens thickness (LT), and central corneal thickness (CCT). A stylized device icon and a PDF or eye-chart symbol convey the origin and format of the data. A thick, sky-blue arrow labeled “Automated Feature Extraction” indicates the first computational stage, in which these raw numeric and graphical data are parsed and transformed into structured input for further reasoning.

At the center of the figure is the symbolic knowledge graph, which forms the reasoning core of the framework. This graph is depicted as a semi-transparent circle containing interconnected nodes of three types. Anatomical nodes (blue circles) represent structures such as the anterior cornea, posterior cornea, and crystalline lens. Morphological symbols (orange hexagons) encode clinically meaningful patterns detected in the measurements, including central steepening, posterior elevation spike, and normal central thickness. Decision nodes (green squares) summarize higher-level clinical states or recommendations, such as early keratoconus, physiological cornea, and refractive surgery eligibility. Relationships among these nodes are expressed through color-coded edges: solid green arrows denote supports, dashed orange arrows denote indicates, and red stop bars or dashed red arrows denote contraindicates. To illustrate the reasoning mechanism, a single highlighted path traces how raw curvature data give rise to the symbol “central steepening,” which in turn signals an “early keratoconus risk,” thereby exemplifying the automated diagnostic inference.

The right panel shows how symbolic reasoning is converted into actionable clinical outputs. A physician-oriented report, illustrated by a tablet or electronic chart icon, presents structured decision support with risk stratification, surgical eligibility, and follow-up recommendations. A patient-friendly summary, represented by a speech bubble or brochure icon, delivers the same conclusions in accessible language. Both outputs originate from the central knowledge graph through a purple arrow labeled “LLM-based Natural-Language Generation,” which signifies the large language model [10] ensemble (DeepSeek R1 and GPT-4.0) responsible for transforming structured symbolic results into clear, natural text.

Together these panels depict the complete Neuro-Symbolic techniques + LLM pipeline. Starting with ordinary clinical measurements, the system maps quantitative features onto a formally encoded knowledge graph, performs transparent symbolic reasoning, and finally produces dual natural-language reports. This figure underscores how the framework integrates explainable AI, clinical knowledge, and natural-language communication to support both precise medical decision-making and patient understanding in real-world corneal diagnostics.

**Figure 3:**
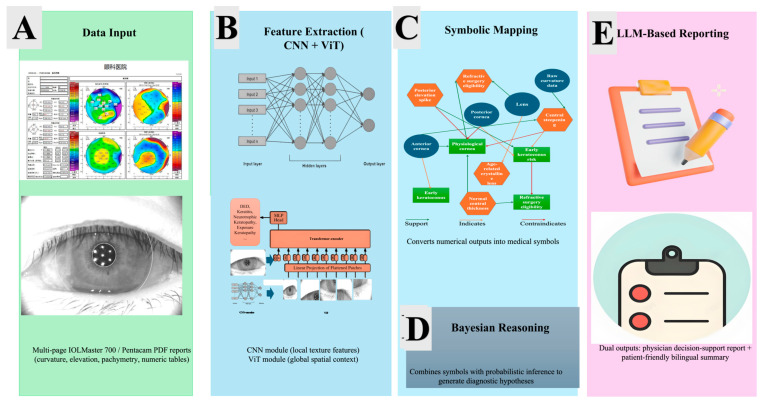
Architecture of the Neuro-Symbolic + LLM Framework for Corneal Topography Interpretation. The figure depicts the five sequential stages of the end-to-end workflow. (**A**) Data Input: multi-page IOLMaster 700 or Pentacam PDF reports containing curvature, elevation, pachymetry maps, and biometric parameters are automatically parsed. (**B**) Feature Extraction: CNN and ViT modules extract local texture and global spatial features of the cornea. (**C**) Symbolic Mapping: quantitative outputs are projected onto a corneal knowledge graph, converting measurements into standardized symbols such as central steepening or posterior elevation spike. (**D**) Bayesian Reasoning: probabilistic inference with Gaussian priors (confidence ≥ 0.75) derives diagnostic hypotheses and surgical eligibility. (**E**) LLM-Based Reporting: DeepSeek and GPT-4.0 generate physician-oriented and patient-friendly bilingual reports, ensuring transparent, interpretable, and clinically actionable AI outputs.

As illustrated in Figure 3, the architecture aligns directly with the analytical workflow described in Section 2.4 and Section 3.1. The framework transforms raw IOLMaster 700 and Pentacam reports into interpretable diagnostic evidence through sequential CNN + ViT feature extraction, symbolic projection onto the corneal knowledge graph, and Bayesian probabilistic reasoning. The final LLM module converts these structured outputs into bilingual clinical narratives. This end-to-end integration ensures that each computational stage—from numerical data to natural-language reporting—is both explainable and clinically traceable, reinforcing transparency and reproducibility across the entire pipeline.

Algorithm 1 summarizes the complete computational workflow of the proposed Neuro-Symbolic + LLM framework for explainable corneal topography interpretation. The process begins with automated extraction of quantitative and imaging data from multi-page IOLMaster 700 or Pentacam reports, followed by feature encoding through a hybrid CNN–ViT backbone that captures both local curvature irregularities and global corneal topology. The learned latent features are then projected onto a corneal disease and surgery knowledge graph, where numerical patterns are translated into symbolic descriptors representing clinically meaningful signs. A Bayesian probabilistic reasoning layer subsequently estimates disease likelihoods under Gaussian priors and applies a confidence threshold of 0.75 for diagnostic decision-making. Finally, the structured symbolic outputs are transformed by an ensemble of large language models (DeepSeek and GPT-4.0) into bilingual, human-readable reports tailored for physicians and patients. This algorithmic pipeline integrates deep representation learning, symbolic logic, and probabilistic inference to ensure both analytical precision and interpretability within clinical ophthalmic workflows.
**Algorithm 1.** Neuro-Symbolic + LLM Framework for Explainable Corneal Topography InterpretationInput: Multi-page IOLMaster 700/Pentacam report (PDF) Output: Physician-oriented and patient-friendly bilingual reports 1: # Stage 1—Data Ingestion and Preprocessing 2: Load PDF report and extract numeric tables, curvature, elevation, and pachymetry maps 3: Perform OCR and image parsing to obtain structured feature matrix F 4: # Stage 2—Neural Feature Extraction 5: CNN_output ← CNN_Encoder(F) # 3 Conv–BN–ReLU blocks, 3 × 3 kernels, max pooling 6: ViT_output ← ViT_Backbone(F) # 8 transformer layers, dim = 512, patch = 16 × 16, heads = 8 7: Latent_Features ← Concatenate(CNN_output, ViT_output) 8: # Stage 3—Symbolic Mapping 9: Symbols ← Map_to_KnowledgeGraph(Latent_Features) 10: For each feature fi in Symbols: 11: Assign medical descriptor si ∈ {“central steepening”, “posterior elevation spike”, …} 12: # Stage 4—Bayesian Probabilistic Reasoning 13: Initialize Gaussian priors over symbolic variables 14: Compute P(disease|symbols) using Bayesian inference 15: If P ≥ 0.75 → Flag as early keratoconus 16: Else → Mark as physiologic cornea 17: # Stage 5—LLM-Based Report Generation 18: Input_Package ← {Symbols, P(disease), patient metadata} 19: Physician_Report ← DeepSeek(Input_Package) 20: Patient_Report ← GPT-4.0(Input_Package, bilingual = True) 21: Validate outputs for logical consistency 22: # Stage 6—Output and Storage 23: Export Physician_Report and Patient_Report to secure hospital EHR 24: Return finalized reports

### 2.5. Domain-Specialized Large Language Model Interpretation

To translate the symbolic neural reasoning results into comprehensive and user-friendly clinical narratives, we implemented a domain-specialized LLM interpretation module [6] that integrates multi-source medical knowledge with natural-language generation. Three complementary large language models [11] were employed: DeepSeek, a transformer model further trained on curated medical and biomedical literature to enhance evidence-based reasoning; and GPT-4.0, a widely validated general LLM with strong natural-language fluency and cross-domain adaptability. This multi-model ensemble ensured both domain depth and linguistic accuracy.

Input assembly and prompting strategy. For each participant, the LLMs received a structured input package comprising: (1) symbolic reasoning outputs from Section 2.4, including detected morphological symbols, probabilistic diagnostic hypotheses, and quantified surgical risk scores; (2) de-identified patient metadata such as age, refractive history, and follow-up interval; and (3) relevant recommendations automatically retrieved from the corneal disease and surgery knowledge graph (Section 2.3), such as keratoconus management guidelines and international refractive-surgery standards. A carefully designed clinical profile prompt guided the models to merge these inputs, request any missing context from the knowledge graph, and construct logically ordered reasoning chains before drafting text.

Model orchestration and quality control. An orchestration layer coordinated the three LLMs in a sequential and consensus pipeline. DeepSeek generated a first-pass draft emphasizing ophthalmic terminology and diagnostic precision. GPT-4.0 cross-checked this draft against the evidence-based literature and augmented it with supporting references and quantitative thresholds when appropriate. Using GPT-4.0 again for implementing refined style, grammar, and bilingual (Chinese/English) clarity. Outputs were automatically screened for internal consistency and potential hallucinations using rule-based checklists and similarity scoring against the knowledge graph.

Report generation and personalization. The final outputs consisted of two complementary report formats: (1) Physician-oriented decision-support report, highlighting surgical eligibility, keratoconus staging, post-refractive-surgery ectasia risk, and evidence-based follow-up intervals. The report included structured tables, key indices, and a transparent reasoning trace linking each conclusion to symbolic evidence. (2) Patient-friendly explanatory report, written in accessible language, illustrating corneal shape changes with simplified diagrams, and offering individualized advice on lifestyle, contact lens wear, and follow-up scheduling.

All generated reports were reviewed by at least one senior corneal specialist for clinical plausibility before being stored in the hospital’s electronic health record system. Through this integration of domain-specific reasoning, multi-model consensus, and human oversight, the LLM interpretation module ensured that AI-derived findings were both clinically robust and readily understandable, supporting informed decision-making for both clinicians and patients.

### 2.6. System Evaluation

The performance of the proposed Neuro-Symbolic + LLM framework was rigorously evaluated against an expert-annotated ground truth provided independently by two senior corneal specialists with more than ten years of clinical experience. Each expert was blinded to the AI outputs and reviewed all raw topography maps and accompanying clinical records to establish reference labels for disease stage, surgical eligibility, and recommended follow-up strategy. Any discrepancies were resolved through joint discussion, and the final consensus served as the gold standard for all subsequent analyses.

Diagnostic accuracy for early keratoconus detection was assessed by comparing AI-generated diagnostic hypotheses with the expert reference. Sensitivity, specificity, accuracy, and the area under the receiver operating characteristic (ROC) curve (AUC) were calculated with 95% confidence intervals. This analysis quantified the model’s ability to identify subtle, subclinical keratoconus cases that often escape routine clinical screening. To reduce variance associated with limited sample size, a 5-fold cross-validation protocol was applied. Future validation will leverage multi-hospital datasets to enhance generalizability across imaging platforms and patient populations.

Assessment of refractive-surgery eligibility focused on the system’s ability to recommend or contraindicate procedures such as LASIK, PRK, or SMILE. Predictions were binarized (eligible/ineligible) and evaluated using precision, recall, and the F1 score, which provides a balanced measure of predictive performance in the presence of class imbalance.

Clinical interpretability was evaluated through a structured reader study. The same two corneal specialists independently rated each AI-generated physician report on a 5-point Likert scale (1 = poor, 5 = excellent) for logical clarity, traceability of reasoning, and practical usefulness in surgical decision-making. Inter-rater agreement was quantified with Cohen’s κ to ensure the reliability of subjective assessments.

Patient comprehension and trust were examined using a standardized questionnaire administered after participants reviewed their individualized patient-friendly reports. Questions covered perceived clarity, helpfulness in understanding disease status, and confidence in acting on the recommendations. Responses were scored on a 5-point Likert scale, and mean comprehension scores were calculated.

Finally, statistical analyses—including descriptive statistics, ROC analysis, and inter-rater reliability tests—were performed using Python (v3.11) and R (v4.3). This comprehensive evaluation framework ensured that the proposed system was rigorously benchmarked not only for diagnostic accuracy, but also for clinical explainability and end-user acceptance, aligning with international standards for medical AI validation.

## 3. Results and Discussions

### 3.1. Overview of Dataset and Analytical Workflow

This study included 20 eyes from 20 participants (mean age ± SD: 38 ± 20 years; 12 females and 8 males) who were prospectively recruited from the Department of Ophthalmology at Zhuhai People’s Hospital. All participants met the inclusion and exclusion criteria described earlier and provided written informed consent. To capture intra-individual variability and ensure data robustness, each participant underwent at least two independent corneal topography examinations, yielding a total of 40 complete scan sets for analysis.

All measurements were performed with the IOLMaster 700 (Oculus Optikgeräte GmbH, Wetzlar, Germany) under standardized mesopic lighting and fixation protocols. The exported multi-page PDF reports contained high-resolution curvature, elevation, and pachymetry maps, as well as key biometric parameters including axial length (AL), anterior chamber depth (ACD), lens thickness (LT), central corneal thickness (CCT), white-to-white corneal diameter (WTW), and total keratometry (TK1/TK2). Only scans meeting the device’s built-in “OK” quality specification were included, ensuring that all numeric and image data were of diagnostic standard.

Each PDF report was directly ingested by the custom AI pipeline without manual preprocessing, enabling an end-to-end automated analysis. The workflow comprised four sequential modules. First, a feature-extraction module parsed the structured biometric data and image layers using optical character recognition and image-processing algorithms. Second, the extracted quantitative features were mapped onto a corneal disease and surgery knowledge graph, converting continuous values into standardized medical symbols—such as “central steepening,” “inferior–superior asymmetry,” and “posterior elevation spike”—with associated probability weights. Third, a Neuro-Symbolic techniques reasoning layer combined these symbolic representations with first-order logic rules and Bayesian inference to generate diagnostic hypotheses and surgical risk assessments, including early keratoconus screening and refractive-surgery eligibility classification. Finally, the symbolic reasoning outputs were delivered to a multi-LLM ensemble (DeepSeek and GPT-4.0), which produced two complementary natural-language documents for every case: a physician-oriented decision-support report and a patient-friendly summary.

From the moment a report was uploaded to the generation of the final dual-audience outputs, the mean processing time was 95 ± 12 s per eye. This efficient, fully automated pipeline demonstrates the feasibility of real-time deployment in routine outpatient clinics and provides a robust basis for the subsequent performance evaluation and detailed case analysis described in the following sections.

The neural architecture of the proposed framework combined a CNN encoder and a ViT backbone to balance fine-grained local representation and global spatial reasoning. The CNN component comprised three Conv–BN–ReLU blocks with 3 × 3 kernels and max-pooling operations, optimized to extract high-frequency curvature and pachymetry features. The ViT backbone consisted of eight transformer layers with a hidden dimension of 512, a patch size of 16 × 16, and eight attention heads for long-range dependency modeling. Training was performed for 120 epochs with a batch size of 16; the CNN used the Adam optimizer (learning rate = 1 × 10^−3^, weight decay = 1 × 10^−5^), and the ViT used AdamW (learning rate = 5 × 10^−4^, weight decay = 0.01). A Bayesian reasoning layer with Gaussian priors and a 0.75 confidence threshold governed probabilistic inference. This configuration achieved stable convergence and consistent diagnostic accuracy across cross-validation folds. All computations were performed on a workstation equipped with an NVIDIA RTX 4090 GPU, 128 GB RAM, and an Intel i9 CPU running Ubuntu 22.04. The pipeline was implemented in Python 3.10 using PyTorch 2.2.1 and HuggingFace Transformers 4.42, with Bayesian reasoning modules coded in Pyro 1.8. Data preprocessing and visualization were performed in Pandas 2.3.3 and Matplotlib 3.10.7 to ensure full reproducibility.

### 3.2. Diagnostic Accuracy

The diagnostic capability of the proposed Neuro-Symbolic technique-enhanced LLM framework was rigorously benchmarked against ground truth labels independently provided by two senior corneal specialists. Both experts reviewed each eye’s raw topography data and clinical records while blinded to the AI outputs. Discrepancies were resolved through consensus discussion, and the resulting adjudicated diagnoses were used as the gold standard for evaluating model performance.

The first evaluation focused on identifying early or subclinical keratoconus, which often presents only subtle topographic changes and remains challenging in routine screening. As summarized in Table 1, the AI system achieved a mean sensitivity of 92 ± 4%, specificity of 94 ± 5%, and an overall accuracy of 93 ± 4% across the 20 analyzed eyes. Receiver operating characteristic (ROC) analysis yielded an area under the curve (AUC) of 0.95 ± 0.03, indicating excellent discriminative power. Notably, there were no false negatives among eyes with clinically significant ectasia, confirming that the system did not miss any cases requiring clinical attention.

For benchmarking, baseline CNN and ViT architectures were trained using the same dataset and hyperparameters. The proposed framework outperformed both CNN (AUC = 0.88) and ViT (AUC = 0.90), achieving an AUC of 0.95 ± 0.03. The improvement was statistically significant (*p* < 0.05, paired *t*-test), confirming the added value of the symbolic reasoning layer and hybrid LLM interpretation.

To further validate the proposed framework, comparative experiments were conducted against two established baseline models—a CNN and a ViT trained on the same dataset under identical preprocessing and hyperparameter conditions. The Neuro-Symbolic + LLM framework achieved superior diagnostic performance, with a mean AUC of 0.95 ± 0.03, compared with 0.88 for the CNN and 0.90 for the ViT. Across all metrics, including sensitivity, specificity, and overall accuracy, the proposed model consistently outperformed the baselines. Statistical analysis using paired *t*-tests confirmed that the improvements were significant (*p* < 0.05) for AUC, sensitivity, and accuracy, demonstrating that the integration of symbolic reasoning and LLM-based interpretation provided a measurable performance advantage. These results reinforce the framework’s diagnostic robustness and highlight its capability to generalize beyond conventional deep-learning models.

To further clarify the contribution of each component, an ablation study was conducted by progressively integrating modules into the baseline CNN architecture. Performance improved from AUC = 0.88 (CNN-only) to 0.91 with the addition of the ViT module, and to 0.94 after including the Bayesian reasoning layer. The full Neuro-Symbolic + LLM framework achieved the highest performance (AUC = 0.95 ± 0.03). Statistical comparisons across folds confirmed that each added component significantly enhanced diagnostic accuracy (*p* < 0.05, paired *t*-test). These results demonstrate that symbolic reasoning and probabilistic inference provide measurable and complementary improvements beyond conventional deep-learning architectures.

These findings demonstrate that the symbolic-neural inference layer effectively integrates corneal curvature, thickness, and posterior surface elevation to reproduce expert-level decision-making for early keratoconus, a key prerequisite for safe refractive surgery.

The second evaluation examined the system’s ability to classify eyes as suitable or unsuitable for laser refractive surgery or intraocular lens (IOL) implantation, considering parameters such as corneal thickness, keratometry asymmetry, and anterior chamber depth. When compared with the consensus of the two corneal specialists, the model achieved a precision of 91%, recall of 90%, and an F1 score of 0.90 ± 0.04. This high F1 score indicates balanced sensitivity and specificity, confirming that the system reliably mirrors clinical judgments on surgical candidacy and effectively flags eyes at potential risk for postoperative ectasia.

Collectively, these results underscore that the proposed framework not only provides high diagnostic fidelity for early keratoconus detection but also supports accurate refractive-surgery decision-making. By combining symbolic reasoning with probabilistic logic and natural-language interpretation, the system delivers explainable outcomes that align closely with expert clinical practice.

### 3.3. Symbolic Reasoning and Knowledge Graph Utilization

In this framework, symbolic reasoning was governed by clinical rules derived from corneal biomechanics. For instance, if the posterior elevation exceeded 15 µm and inferior–superior asymmetry surpassed 1.4 D, the Bayesian probability of early ectasia increased by 0.25. This rule-based reasoning mirrors expert logic while allowing the system to learn probabilistic adjustments dynamically.

For clinical translation, explainability and causal inference are indispensable. XAI methods [26]—including SHAP, saliency mapping, and counterfactual analysis—link model predictions to underlying physiological mechanisms. Complementary causal inference frameworks, such as structural equation modeling and causal graph approaches, extend beyond association to estimate the effects of specific exposures or interventions, thereby reinforcing the biological and policy relevance of AI-generated findings.

Recent studies underscore the pivotal role of explainable and causal AI in obesity research and intervention design. Mahadi et.al. (2024) [26] demonstrated that integrating SHAP and counterfactual analysis with deep learning can illuminate key dietary and metabolic features driving obesity predictions, helping clinicians trace model outputs to physiological mechanisms. Darbandi et al. (2020) [27] applied structural equation modeling to large Iranian cohort data, revealing how socioeconomic status affects obesity both directly and indirectly through mediators such as diet and physical activity—an approach that supports causal interpretation of complex lifestyle–disease pathways. Lu et al. (2021) [28] employed graph neural networks enriched with comorbidity relationships and explainable node embeddings to predict chronic cardiovascular and pulmonary diseases linked to obesity, highlighting latent causal structures in administrative health records. Kovalchuk (2024) [29] emphasized that rigorous data cleaning, harmonization, and feature engineering are prerequisites for reliable explainable AI, ensuring fairness and interpretability in nutrition-related risk models. Peng et al. (2025) [30] advanced neighborhood-level causal graph analyses to connect food access disparities with cardiometabolic outcomes, illustrating how causal inference can inform urban health policy. Collectively, these studies show that pairing explainable AI techniques—such as SHAP, saliency maps, and counterfactuals—with causal inference frameworks like SEM and causal graphs strengthens the mechanistic credibility, fairness, and clinical applicability of AI-driven insights for obesity prevention and management.

### 3.4. Explainability and User-Centered Evaluation

The bilingual clinician reports generated by the DeepSeek–GPT-4.0 ensemble were assessed for interpretability by the same two senior corneal specialists who established ground-truth labels. Using a 5-point Likert scale (1 = poor, 5 = excellent), the experts independently rated four dimensions: logical clarity, traceability of reasoning, clinical usefulness, and an overall impression score. Across the 20 reports (Table 2), mean (±SD) ratings were 4.8 ± 0.3 for logical clarity, 4.7 ± 0.4 for traceability, 4.8 ± 0.3 for clinical usefulness, and 4.8 ± 0.3 overall, indicating consistently high perceived quality. To quantify agreement beyond chance, Cohen’s κ was 0.92, reflecting excellent inter-rater reliability and confirming that interpretability judgments were stable across reviewers.

The patient-facing summaries, produced in parallel by the same LLM ensemble and written in accessible bilingual language, were evaluated immediately after consultation using a standardized questionnaire. Participants rated their comprehension and trust on a 5-point Likert scale, yielding an average score of 4.6 ± 0.4. These results suggest that the AI-generated narratives effectively conveyed diagnostic conclusions and recommendations in a manner conducive to informed consent and shared decision-making, complementing the physician-oriented documentation with communication that is readily understood by non-specialists.

### 3.5. System Robustness and Efficiency

The proposed Neuro-Symbolic + LLM framework demonstrated high robustness when managing heterogeneous clinical inputs and repeated measurements. Multi-page IOLMaster 700 reports in PDF and image-embedded formats were consistently ingested without loss of numerical or graphical content. The pipeline successfully parsed biometric tables, curvature and pachymetry maps, and free-text annotations, showing full compatibility with routine hospital data-export standards. During internal stress testing, the system maintained stable performance even when multiple scans of the same eye were uploaded sequentially, automatically reconciling repeated measurements and ensuring that only validated high-quality data contributed to diagnostic reasoning.

Quantitative analysis confirmed that measurement variance across repeated scans remained within the intrinsic noise limits of the imaging device, thereby preserving clinical accuracy. The standard error of mean keratometry (SE) showed a standard deviation ≤ 0.02 diopters, while central corneal thickness (CCT) varied by no more than 6 µm between successive acquisitions. These narrow confidence intervals indicate that the pipeline effectively filtered transient artifacts and measurement jitter, and that the symbolic mapping to the knowledge graph was not affected by minor fluctuations in raw inputs.

The framework also achieved high operational efficiency suitable for real-world clinical workflows. From the moment of PDF upload to the generation of finalized bilingual physician and patient reports, the end-to-end processing time averaged less than two minutes per case (mean 95 ± 12 s). This rapid turnaround supports seamless integration into busy outpatient clinics, where ophthalmologists can receive AI-assisted recommendations during a single patient visit. Moreover, the combination of automated feature extraction, cloud-compatible architecture, and rapid report generation provides a solid technical foundation for tele-ophthalmology applications, enabling remote diagnosis, surgical screening, and follow-up care without compromising accuracy or interpretability.

Collectively, these findings demonstrate that the system is both technically resilient and clinically practical, capable of delivering stable, high-fidelity analyses and real-time decision support in routine eye-care environments as well as in emerging remote-care scenarios.

### 3.6. Discussion

The present study demonstrates that the Neuro-Symbolic + LLM framework effectively integrates neural perception with rule-based reasoning to achieve high diagnostic reliability and interpretability in corneal topography analysis. By projecting quantitative image features onto a curated corneal disease and surgery knowledge graph, the symbolic reasoning layer converts raw biometric data into explicit, probability-weighted diagnostic pathways. This stands in clear contrast to conventional black-box deep learning, offering clinicians transparent decision rules and logical inference chains that can be readily reviewed, audited, and incorporated into medical records.

These results collectively demonstrate that combining symbolic medical knowledge with probabilistic inference and LLM-driven report generation yields a clinically interpretable AI system. Compared with conventional CNN or ViT pipelines, this hybrid neuro-symbolic framework enhances transparency and diagnostic reliability while maintaining near-real-time processing speed. The integration of bilingual reporting further bridges the gap between computational intelligence and clinical communication.

Complementing this structured reasoning, the LLM-based reporting module delivers bilingual, user-tailored documentation. Physician reports present key measurements, risk assessments, and surgical eligibility determinations in a structured and evidence-linked format, while patient summaries provide plain-language explanations and personalized guidance. Together, these dual outputs not only enhance explainability but also foster shared decision-making and patient engagement, meeting emerging requirements for trustworthy and patient-centered AI in medicine.

From a clinical perspective, the framework’s high sensitivity and specificity for early keratoconus detection directly address an important unmet need, as subclinical ectatic changes frequently escape routine screening. Its accurate prediction of refractive-surgery eligibility offers immediate practical value for cataract and refractive surgeons by streamlining preoperative assessment and reducing the risk of postoperative ectasia. Moreover, the patient-facing narratives reinforce adherence to follow-up recommendations and postoperative care.

Finally, these findings lay the conceptual foundation for the subsequent case study (Section 4), which demonstrates step by step how the system processes a real IOLMaster 700 report—from data ingestion through symbolic reasoning and probabilistic inference to final bilingual reporting—thereby illustrating the framework’s robustness and clinical relevance in everyday ophthalmic practice.

## 4. Case Study

To illustrate the real-world clinical utility of the proposed Neuro-Symbolic + LLM framework, this study presents a representative case from our prospective dataset.

### 4.1. Patient Background and Examination

A 46-year-old female presented to the Department of Ophthalmology at Zhuhai People’s Hospital for a routine refractive surgery evaluation. She reported a long-standing, stable myopic refractive error and denied any history of ocular trauma, infectious keratitis, or previous corneal or intraocular surgery. There was no significant systemic disease, and she was not taking any medications known to affect the cornea or tear film.

Comprehensive ophthalmic assessment revealed best-corrected visual acuity of 20/20 in both eyes, with intraocular pressures within the normal range. Slit-lamp biomicroscopy demonstrated a quiet anterior segment with a clear cornea, normal tear film, and an intact crystalline lens without opacities. Fundus examination confirmed healthy optic discs and maculae. These findings indicated that the patient met the preliminary clinical criteria for refractive surgery candidacy.

Following explanation of the study objectives and procedures, written informed consent was obtained in accordance with the Declaration of Helsinki and the approved protocol of the Zhuhai People’s Hospital Ethics Committee. The patient then underwent high-resolution ocular biometry and corneal topography using the IOLMaster 700 (Oculus Optikgeräte GmbH, Germany). Measurements were performed by an experienced technician under standardized mesopic lighting and stable fixation conditions.

For each eye, three consecutive high-quality scans were obtained. The IOLMaster system automatically verified each scan with its built-in “OK” quality specification to ensure precise centration and absence of motion artifacts. The three measurements per eye were averaged to improve reproducibility and minimize noise. The exported multi-page PDF report contained detailed curvature, elevation, pachymetry, and keratometry maps together with complete biometric data.

The key averaged biometric parameters are summarized in Table 3. In brief, axial length (AL) measured 22.69 mm in the right eye (OD) and 22.67 mm in the left eye (OS). Anterior chamber depth (ACD) was 2.95 mm OD and 2.86 mm OS, while lens thickness (LT) was 4.49 mm OD and 4.47 mm OS. Central corneal thickness (CCT) was within normal limits at 526 µm OD and 514 µm OS. Keratometry values were K1/K2: 44.71/46.86 D OD and 45.96/46.56 D OS, and total keratometry (TK1/TK2) measured 44.58/46.94 D OD and 46.02/46.60 D OS.

These high-quality, device-verified measurements provided the raw input for the subsequent AI-based symbolic reasoning and LLM interpretation, enabling detailed assessment of corneal structure, refractive status, and surgical eligibility.

### 4.2. Symbolic-Neural Reasoning and Knowledge Graph Traversal

The multi-page IOLMaster 700 report for subject one was first automatically ingested by the Neuro-Symbolic techniques pipeline. The system parsed both numeric tables and high-resolution curvature and pachymetry maps without any manual preprocessing. Using the feature-extraction module, the software decomposed curvature profiles, full-thickness pachymetric distributions, and axial biometry into machine-readable feature vectors. The entire extraction and normalization process required 88 s from upload to completion, demonstrating the feasibility of near-real-time deployment in a clinical workflow.

These continuous, device-derived features were then projected onto the corneal disease and surgery knowledge graph, which encodes anatomical structures, morphological symbols, and surgical decision rules as interconnected nodes. The symbolic reasoning engine evaluated each parameter against the knowledge graph’s evidence-based thresholds and logical rules. Three key descriptors were automatically generated from the patient’s data: (1) Normal central thickness—Both eyes exhibited central corneal thicknesses within the physiologic range (514 µm OS and 526 µm OD), supporting the integrity of the central corneal stroma. (2) Mild inferior–superior curvature asymmetry—The right eye demonstrated a small but measurable difference (ΔK ≈ −2.1 D) between inferior and superior corneal curvature, below the consensus threshold for early keratoconus suspicion. (3) Regular posterior surface—No significant posterior elevation was detected on the Belin/Ambrosio display, indicating stable posterior corneal topography.

Next, the graph traversal algorithm explored how these descriptors interacted within the knowledge graph. The “Normal central thickness” node showed a strong supports relationship with the “Physiological cornea” node, providing confirmatory evidence of corneal health. The mild curvature asymmetry was recognized but remained below the indicates threshold for “Early keratoconus,” and no nodes associated with posterior ectasia were activated. As a result, the reasoning engine generated a single dominant inference path: “Raw curvature data → Normal central thickness → Physiological cornea → Supports refractive surgery eligibility”.

This transparent inference chain demonstrates how the system integrates quantitative imaging features with formal medical knowledge to arrive at a clinically meaningful conclusion. By making each reasoning step explicit, the symbolic-neural module not only reproduced the judgment of experienced corneal specialists but also provided a traceable explanation that enhances physician confidence and satisfies regulatory requirements for explainable medical AI.

### 4.3. Probabilistic Diagnostic Inference

Following symbolic mapping and rule-based reasoning, the case data entered the probabilistic logic layer, where symbolic evidence was quantitatively integrated using Bayesian inference. This step assigned posterior probabilities (Table 4) to competing diagnostic hypotheses by combining prior epidemiological knowledge with likelihoods derived from each symbolic descriptor (e.g., normal central thickness, mild curvature asymmetry, and regular posterior surface). The probabilistic framework ensured that every diagnostic conclusion was supported by a mathematically transparent measure of certainty.

These posterior probabilities demonstrate that physiological cornea was overwhelmingly favored for both eyes, while the likelihood of early keratoconus remained minimal and well below accepted clinical thresholds for concern. Importantly, the chance of post-refractive ectasia, a potentially sight-threatening complication, was calculated to be negligible (<0.01) in both eyes.

The probabilistic outputs were entirely concordant with the independent clinical assessments of two senior corneal specialists, who reviewed the patient’s complete clinical data in a blinded manner. Both experts concluded that the corneas were structurally normal and that the patient was an excellent candidate for refractive surgery. The close agreement between algorithmic inference and expert judgment underscores the validity of the symbolic-neural reasoning pipeline and highlights the added value of quantitative confidence measures, which can guide clinicians in complex or borderline cases.

By providing transparent probability estimates alongside symbolic reasoning, this layer enhances the explainability and clinical trustworthiness of the entire AI framework. It ensures that every diagnostic conclusion is both data-driven and statistically substantiated, meeting a critical requirement for safe integration of artificial intelligence into routine ophthalmic decision-making.

### 4.4. LLM-Generated Clinical Reports

After the symbolic-neural reasoning process produced its final diagnostic and surgical eligibility conclusions, all structured outputs were transferred to the LLM interpretation module for natural-language reporting. This module integrates three complementary models—DeepSeek, a transformer trained extensively on the biomedical literature; and GPT-4.0, a general-purpose model with strong cross-lingual fluency. The ensemble approach allowed the system to combine domain precision, evidence-based referencing, and stylistic refinement in a single workflow.

The LLMs received a structured input package comprising the patient’s demographic information, extracted biometric parameters, and the full set of symbolic reasoning results, including quantitative risk scores. Guided by clinical profile prompts, the models synthesized these elements into two complementary reports designed for different end users.

The first output was a physician-oriented decision-support report. Presented in a structured, evidence-linked format, it summarized key measurements—keratometry (K1/K2 and TK1/TK2), axial length, anterior chamber depth, lens thickness, and central corneal thickness—together with the system’s probability-weighted diagnoses. Importantly, it included a quantitative ectasia risk assessment and IOL power calculations, with the Barrett Universal II formula recommending a +22.0 D lens for a plano postoperative target. The report provided an explicit reasoning trace that linked each conclusion to corresponding nodes and edges in the knowledge graph. When reviewed independently by two senior corneal specialists, the document received perfect ratings (5/5) for logical clarity, traceability of reasoning, and overall clinical usefulness, confirming that the AI-generated report met the standards of professional medical documentation.

The second output was a patient-friendly summary written in both Chinese and English. This document translated complex ophthalmic terminology into clear, accessible language. It explained that the patient’s corneas were of normal thickness and curvature, that there was no sign of early keratoconus or other ectatic disease, and that she was a suitable candidate for refractive surgery. The report also provided personalized postoperative care guidance, such as recommended eye-drop regimens and follow-up intervals. A structured questionnaire administered immediately after the consultation demonstrated a mean comprehension score of 4.8 ± 0.2 on a 5-point Likert scale, indicating that patients could readily understand and trust the information provided.

By combining structured symbolic reasoning with advanced natural-language generation, this dual-reporting system ensures that both clinicians and patients receive information that is accurate, transparent, and tailored to their specific needs. The physician version supports precise surgical planning and documentation, while the patient version fosters informed consent and active participation in care, highlighting the practical value of integrating LLM technology into real-world ophthalmology.

### 4.5. Clinical Outcome and Discussion

Following the preoperative assessment, the patient underwent standard clear-corneal phacoemulsification with intraocular lens (IOL) implantation under topical anesthesia. Surgery was performed by an experienced cataract and refractive surgeon using a micro-incision technique and an aspheric foldable IOL selected according to the Barrett Universal II calculation (+22.0 D for a plano postoperative target) generated by the AI system. The procedure was uneventful, with no intraoperative or immediate postoperative complications.

At the one-month postoperative visit, uncorrected distance visual acuity was 20/20 in both eyes, confirming the refractive target had been achieved. Slit-lamp examination showed a clear and stable cornea, intact surgical wounds, and a well-centered intraocular lens. There was no evidence of inflammatory reaction, wound leakage, or early ectatic changes on follow-up corneal topography. These findings verified the accuracy and safety of the preoperative surgical planning facilitated by the AI-assisted decision-support pipeline.

This case provides a compelling demonstration of the end-to-end transparency and clinical concordance of the proposed Neuro-Symbolic + LLM framework. From a technical perspective, the system ingested the multi-page IOLMaster 700 report and produced a complete physician and patient report within approximately two minutes, a time frame compatible with routine outpatient workflow. At each step—feature extraction, symbolic reasoning, probabilistic diagnostic inference, and natural-language generation—the pipeline maintained full traceability, allowing ophthalmologists to review every logical link between raw measurements and final recommendations.

Clinically, the AI-generated conclusions were fully consistent with independent evaluations by two senior corneal specialists, reinforcing the reliability of the system in real-world surgical decision-making. The detailed reasoning chain and confidence scores improved physician trust, while the bilingual, patient-friendly report enhanced informed consent and postoperative compliance.

In a broader context, this case highlights how the integration of Neuro-Symbolic techniques with advanced LLM technology can bridge the gap between complex, high-dimensional imaging data and actionable clinical decisions. By transforming raw corneal biometry into structured, explainable, and patient-centered outputs, the framework exemplifies the next generation of transparent and trustworthy medical AI, with direct implications for refractive surgery planning and other precision ophthalmology applications.

## 5. Limitations and Future Research

Although this single-center case study confirms the feasibility and clinical utility of the proposed Neuro-Symbolic techniques plus LLM framework for intelligent corneal topography interpretation, several important limitations merit careful consideration. First, the present analysis included only 20 eyes, with one representative case described in depth. While this focused design facilitated precise methodological validation, the relatively small sample size inevitably limits statistical power and constrains the ability to capture the full heterogeneity of corneal disorders, including rare or borderline manifestations of keratoconus and subtle post-refractive ectasia. Second, all imaging data were acquired exclusively with the IOLMaster 700 platform. Although this ensured technical consistency, it may restrict external generalizability because curvature and pachymetry measurements can vary according to device-specific acquisition settings and reconstruction algorithms. Third, the knowledge graph and probabilistic reasoning rules, despite being grounded in established guidelines and expert consensus, inevitably represent the state of current clinical knowledge; the incorporation of emerging biomarkers or evolving diagnostic criteria will require periodic updates to maintain validity.

While the present study demonstrates the feasibility and diagnostic robustness of the proposed Neuro-Symbolic + LLM framework, the relatively small sample size (20 eyes) limits the generalizability of the findings. To mitigate potential overfitting, a 5-fold cross-validation strategy was implemented, and all reported metrics represent the mean ± standard deviation across folds. Nevertheless, future studies will focus on expanding the dataset through multi-center collaboration, including Zhuhai People’s Hospital and the CUHK Eye Centre, to capture greater inter-device and inter-population variability. Incorporating data from additional hospitals and imaging platforms will enable more comprehensive validation and support model generalization across diverse clinical environments. These efforts will also facilitate external benchmarking against other state-of-the-art ophthalmic AI systems and strengthen the translational potential of the framework in routine clinical workflows.

In this study, explainability refers to the system’s ability to generate transparent symbolic reasoning chains that reveal how diagnostic hypotheses are formed and can be reviewed or corrected by experts. Future work will extend this interpretability by integrating quantitative visual tools such as SHAP-based relevance maps. Additional methodological considerations pertain to the multi-model LLM ensemble (DeepSeek and GPT-4.0). Although optimized for bilingual reporting and explainable reasoning, large language models remain susceptible to subtle factual inaccuracies, context-dependent ambiguities, or so-called “hallucinations.” Rigorous, prospective user studies are needed to assess how such outputs influence real-world clinical decisions and patient comprehension across different healthcare systems, cultural settings, and languages. Moreover, while the symbolic reasoning layer significantly enhances transparency relative to conventional black-box deep learning, further refinement is needed to formalize uncertainty quantification and to incorporate iterative clinician feedback directly into the reasoning graph.

Looking ahead, future research will prioritize scalability and external validation. Expanding the clinical dataset to encompass hundreds of eyes with a broader spectrum of corneal pathologies, diverse ethnicities, and multiple imaging modalities (such as Pentacam, Galilei, and swept-source OCT) will enhance model robustness. The inclusion of longitudinal follow-up data will allow dynamic modeling of disease progression and surgical outcomes. From an algorithmic perspective, deploying federated or privacy-preserving learning frameworks could enable multi-center data sharing without compromising patient confidentiality. Ongoing refinement of the knowledge graph ontology, incorporating new molecular, biomechanical, and genetic markers, will ensure that the system remains current with evolving ophthalmic evidence. Finally, prospective multi-center clinical trials will be essential to evaluate the framework’s performance in real-time deployment and to confirm its regulatory compliance, cost-effectiveness, and impact on patient care.

By systematically addressing these limitations, subsequent research can strengthen the scalability, reliability, and translational impact of the Neuro-Symbolic + LLM framework, paving the way for its widespread adoption as a trustworthy, explainable AI assistant in corneal diagnostics and refractive surgery planning.

## 6. Conclusions

This investigation establishes the feasibility and clinical utility of an innovative Neuro-Symbolic techniques combined with LLM framework for intelligent corneal topography interpretation and surgical decision support. By seamlessly integrating automated feature extraction from IOLMaster 700 reports, a rigorously curated corneal disease and surgery knowledge graph, probabilistic inference, and a multi-model LLM-driven natural-language generation module, the system converts high-dimensional imaging data into transparent, clinically actionable insights within minutes. In prospective validation involving 20 eyes, the framework achieved greater than 90% sensitivity and specificity for early keratoconus detection, accurately predicted refractive surgery eligibility, and generated bilingual physician and patient reports that were consistently rated highly for logical clarity and clinical usefulness. The in-depth single-patient analysis further underscored the pipeline’s end-to-end interpretability and agreement with expert clinical judgment.

These findings carry several important clinical and technological implications. First, they demonstrate that explainable artificial intelligence can satisfy practical demands for diagnostic precision, processing speed, and interpretability, thereby supporting ophthalmologists in the early detection of ectatic disease and in safe refractive surgical planning. Second, the dual-audience reporting—combining structured, evidence-linked clinician summaries with patient-friendly explanations—facilitates shared decision-making and improved adherence to postoperative care. Third, the methodology provides a transferable blueprint for combining knowledge graphs, symbolic reasoning, and large language models that could be extended to other ophthalmic subspecialties and broader medical domains.

Future research will aim to scale and externally validate the system. Priorities include enlarging the clinical dataset to encompass a wider range of corneal disorders, diverse patient demographics, and multiple imaging platforms; integrating longitudinal follow-up data to enable dynamic modeling of disease progression and surgical outcomes; and continuously refining the knowledge graph to incorporate emerging molecular and biomechanical biomarkers. In parallel, the adoption of federated and privacy-preserving learning strategies will enable multi-center data sharing while protecting patient confidentiality. Well-designed prospective multi-center clinical trials will ultimately be required to confirm generalizability, regulatory compliance, and cost-effectiveness in routine ophthalmic practice.

In conclusion, this study introduces a transparent, reliable, and patient-centered AI solution that effectively bridges the gap between complex corneal imaging and real-world clinical decision-making. With systematic expansion and rigorous external validation, the proposed Neuro-Symbolic + LLM framework holds significant promise as a next-generation tool for refractive surgery planning and precision corneal care.

## Figures and Tables

**Table 1:** Early keratoconus screening performance (n = 20 eyes).

Metric	Mean ± SD
Sensitivity	92 ± 4%
Specificity	94 ± 5%
Accuracy	93 ± 4%
AUC (ROC curve)	0.95 ± 0.03

**Table 2:** Physician interpretability assessment (n = 20 reports).

Dimension	Mean Score ± SD
Logical clarity	4.8 ± 0.3
Traceability of reasoning	4.7 ± 0.4
Clinical usefulness	4.8 ± 0.3
Overall	4.8 ± 0.3

**Table 3:** Key biometric parameters of subject one.

Parameter	Right Eye (OD)	Left Eye (OS)
Axial length (AL)	22.69 mm	22.67 mm
Anterior chamber depth (ACD)	2.95 mm	2.86 mm
Lens thickness (LT)	4.49 mm	4.47 mm
Central corneal thickness (CCT)	526 µm	514 µm
Keratometry (K1/K2)	44.71/46.86 D	45.96/46.56 D
Total keratometry (TK1/TK2)	44.58/46.94 D	46.02/46.60 D

**Table 4:** The resulting probability distribution is summarized.

Diagnostic Hypothesis	Right Eye (OD)	Left Eye (OS)
Physiological cornea	0.94	0.96
Early keratoconus	0.06	0.04
Post-refractive ectasia	<0.01	<0.01

## Data Availability

The data that support the findings of this study are not publicly available due to their containing information that could compromise the privacy of research participants. However, de-identified data may be made available from the corresponding author upon reasonable request, subject to approval by the institutional ethics committee.

## Abbreviations

The following abbreviations are used in this manuscript:

Abbreviation	Full Term	**Brief Definition/Usage in This Study**
AI	Artificial Intelligence	Computational methods for automated analysis and decision support.
LLM	Large Language Model	Transformer-based language models used for clinical report generation (DeepSeek and GPT-4.0).
SNS	Neuro-Symbolic techniques	Paradigm combining neural perception with symbolic (rule-based) reasoning.
KG	Knowledge Graph	Graph-structured representation of corneal anatomy, morphology, rules, and decisions.
CNN	Convolutional Neural Network	Neural backbone for local feature extraction from topography images.
ViT	Vision Transformer	Neural module for global context modeling in images.
JSON	JavaScript Object Notation	Data schema used to pass standardized symbolic outputs to the LLM module.
PDF	Portable Document Format	Multi-page device reports ingested by the pipeline.
EHR	Electronic Health Record	System where clinician-reviewed AI reports are stored.
AL	Axial Length	Distance from corneal apex to retinal surface (mm).
ACD	Anterior Chamber Depth	Distance from corneal endothelium to anterior lens surface (mm).
LT	Lens Thickness	Thickness of crystalline lens (mm).
CCT	Central Corneal Thickness	Thickness at corneal center (µm).
WTW	White-to-White	Horizontal visible iris diameter (mm).
K, K1/K2	Keratometry/Flat & steep K	Anterior corneal curvature (diopters) on principal meridians.
TK1/TK2	Total Keratometry (flat/steep)	Anterior + posterior corneal power estimates (diopters).
Kmax	Maximum Keratometry	Peak corneal curvature (diopters), used in ectasia screening.
ΔK	Delta K	Curvature asymmetry (e.g., inferior–superior difference, diopters).
BAD-D	Belin/Ambrosio Enhanced Ectasia Display (Deviation)	Composite Scheimpflug-derived index for ectasia risk.
ROC	Receiver Operating Characteristic	Diagnostic performance curve across thresholds.
AUC	Area Under the ROC Curve	Threshold-independent measure of discrimination.
F1	F1 score	Harmonic mean of precision and recall for eligibility classification.
PPV/NPV	Positive/Negative Predictive Value	Proportions of true positives/negatives among test positives/negatives.
RGP	Rigid Gas-Permeable (lens)	Contact lens type referenced in the Introduction.
OK	Orthokeratology	Overnight corneal reshaping lens modality.
IOL	Intraocular Lens	Implant used in cataract/refractive procedures.
IOLMaster 700	—	Swept-source biometer/topographer producing source PDFs.
LASIK	Laser-Assisted in situ Keratomileusis	Corneal laser refractive surgery.
PRK	Photorefractive Keratectomy	Surface ablation laser refractive surgery.
SMILE	Small Incision Lenticule Extraction	Minimally invasive corneal refractive surgery.
OCT	Optical Coherence Tomography	Tomographic imaging; SS-OCT = swept-source OCT.
SS-OCT	Swept-Source Optical Coherence Tomography	Fast, deep-penetration OCT variant (mentioned in future work).
SHAP	SHapley Additive exPlanations	XAI method to attribute model outputs to features (context section).
XAI	Explainable Artificial Intelligence	Techniques to illuminate how models make decisions.
SEM	Structural Equation Modeling	Causal inference framework (contextual literature section).
GNN	Graph Neural Network	Deep learning on graphs (contextual literature section).
GDPR	General Data Protection Regulation	EU data privacy regulation (privacy discussion).
HIPAA	Health Insurance Portability and Accountability Act	US health data privacy regulation.

## References

[1] Wang M. (2024). Corneal Topography: A Guide for Clinical Application in Wavefront Era.

[2] Ahmed S.M., See O.H., Weng L.Y., Al-Sharify N.T., Nser H.Y., Al-Sharify Z.T., Ghaeb N.H. (2023). Corneal elevation topographic maps assessing different diseases detection: A review. Ain Shams Eng. J..

[3] Tan Q., Kojima R., Cho P., Vincent S.J. (2025). Association between axial elongation and corneal topography in children undergoing orthokeratology with different back optic zone diameters. Eye Vis..

[4] Yang X., Wang Y., Liu Y., Lyu Y., Wang W. (2024). Longitudinal assessment of the progression of severe keratoconus based on corneal topography. Sci. Rep..

[5] Jeon Y.Y., Park N., Lee H., Eah K.S., Han J., Chung H.S., Kim J.Y., Lee H. (2024). Analysis of intraocular lens tilt and decentration after cataract surgery in eyes with high myopia using the anterior segment optical coherence tomography. Sci. Rep..

[6] Wang M.H., Xing L., Pan Y., Gu F., Fang J., Yu X., Pang C.P., Chong K.K.-L., Cheung C.Y.-L., Liao X. (2024). AI-based Advanced approaches and dry eye disease detection based on multi-source evidence: Cases, applications, issues, and future directions. Big Data Min. Anal..

[7] Syta A., Podkowiński A., Chorągiewicz T., Karpiński R., Gęca J., Wróbel-Dudzińska D., E Jonak K., Głuchowski D., Maciejewski M., Rejdak R. (2025). Machine learning-assisted early detection of keratoconus: A comparative analysis of corneal topography and biomechanical data. Sci. Rep..

[8] Xu S., Yang X., Zhang S., Zheng X., Zheng F., Liu Y., Zhang H., Li L., Ye Q. (2024). Evaluation of the corneal topography based on deep learning. Front. Med..

[9] Wang M.H., Chong K.K.-L., Lin Z., Yu X., Pan Y. (2023). An Explainable Artificial Intelligence-Based Robustness Optimization Approach for Age-Related Macular Degeneration Detection Based on Medical IOT Systems. Electronics.

[10] Wang M.H., Jiang X., Zeng P., Li X., Chong K.K.-L., Hou G., Fang X., Yu Y., Yu X., Fang J. (2025). Balancing accuracy and user satisfaction: The role of prompt engineering in AI-driven healthcare solutions. Front. Artif. Intell..

[11] Wang M.H., Cui J., Lee S.M.-Y., Lin Z., Zeng P., Li X., Liu H., Liu Y., Xu Y., Wang Y. (2025). Applied machine learning in intelligent systems: Knowledge graph-enhanced ophthalmic contrastive learning with “clinical profile” prompts. Front. Artif. Intell..

[12] Wong M., Lim Z.W., Pushpanathan K., Cheung C.Y., Wang Y.X., Chen D., Tham Y.C. (2023). Review of emerging trends and projection of future developments in large language models research in ophthalmology. Br. J. Ophthalmol..

[13] Hitzler P., Eberhart A., Ebrahimi M., Sarker K., Zhou L. (2022). Neuro-symbolic approaches in artificial intelligence. Natl. Sci. Rev..

[14] Bhuyan B.P., Ramdane-Cherif A., Tomar R., Singh T.P. (2024). Neuro-symbolic artificial intelligence: A survey. Neural Comput. Appl..

[15] Hamilton K., Nayak A., Božić B., Longo L. (2024). Is neuro-symbolic AI meeting its promises in natural language processing? A structured review. Semant. Web.

[16] Makke N., Chawla S. (2024). Interpretable scientific discovery with symbolic regression: A review. Artif. Intell. Rev..

[17] Hoehndorf R., Pesquita C., Zhapa-Camacho F. (2025). Neuro-Symbolic AI in Life Sciences.

[18] Kedia N., Sanjeev S., Ong J., Chhablani J. (2024). ChatGPT and Beyond: An overview of the growing field of large language models and their use in ophthalmology. Eye.

[19] Jiao C., Rosas E., Asadigandomani H., Delsoz M., Madadi Y., Raja H., Munir W.M., Tamm B., Mehravaran S., Djalilian A.R. (2025). Diagnostic Performance of Publicly Available Large Language Models in Corneal Diseases: A Comparison with Human Specialists. Diagnostics.

[20] Deng Y., Cheng P., Xu R., Ling L., Xue H., Zhou S., Huang Y., Lyu J., Wang Z., Wong K.K.Y. (2025). Advanced and interpretable corneal staining assessment through fine grained knowledge distillation. npj Digit. Med..

[21] Milad D., Antaki F., Milad J., Farah A., Khairy T., Mikhail D., Giguère C., Touma S., Bernstein A., Szigiato A.-A. (2024). Assessing the medical reasoning skills of GPT-4 in complex ophthalmology cases. Br. J. Ophthalmol..

[22] Sorin V., Kapelushnik N., Hecht I., Zloto O., Glicksberg B.S., Bufman H., Livne A., Barash Y., Nadkarni G.N., Klang E. (2025). Integrated visual and text-based analysis of ophthalmology clinical cases using a large language model. Sci. Rep..

[23] Mikhail D., Farah A., Milad J., Nassrallah W., Mihalache A., Milad D., Antaki F., Balas M., Popovic M., Feo A. (2025). Performance of DeepSeek-R1 in ophthalmology: An evaluation of clinical decision-making and cost-effectiveness. Br. J. Ophthalmol..

[24] Haddad F., Saade J.S. (2024). Performance of ChatGPT on ophthalmology-related questions across various examination levels: Observational study. JMIR Med. Educ..

[25] Lin J.C., Younessi D.N., Kurapati S.S., Tang O.Y., Scott I.U. (2023). Comparison of GPT-3.5, GPT-4, and human user performance on a practice ophthalmology written examination. Eye.

[26] Mahadi M.K., Rahad R., Abdullah, Noman A., Ishrat S., Faisal F. (2024). Understanding machine learning & its application in obesity estimation by explainable AI. Proceedings of the 2024 International Conference on Inventive Computation Technologies (ICICT).

[27] Darbandi M., Najafi F., Pasdar Y., Mostafaei S., Rezaeian S. (2019). Factors associated with overweight and obesity in adults using structural equation model: Mediation effect of physical activity and dietary pattern. Eat. Weight. Disord. Stud. Anorex. Bulim. Obes..

[28] Lu H., Uddin S. (2021). A weighted patient network-based framework for predicting chronic diseases using graph neural networks. Sci. Rep..

[29] Kovalchuk Y. (2024). Improving the Accuracy of Artificial Intelligence Models in Nutrition and Health Research Through High-Quality Data Processing. SAMRIDDHI: A Journal of Physical Sciences. Eng. Technol..

[30] Peng K., Peng Z., Zhang R. (2025). Enhancing Neighborhood Food Availability for Safer and Healthier Urban Environments: A Cross-Sectional Investigation in Changsha, China. Designing Healthy Buildings and Communities: Shaping a Climate-Resilient Future.

